# Preventive Strategies to Reduce Sarcopenia Risk During GLP-1-Based Anti-Obesity Therapy

**DOI:** 10.3390/jcm15155870

**Published:** 2026-07-27

**Authors:** Andrej Belančić, Kristina Skroče, Elvira Meni Maria Gkrinia, Mihaela Marinović Glavić, Man Ki Kwok

**Affiliations:** 1 Department of Basic and Clinical Pharmacology and Toxicology, Faculty of Medicine, University of Rijeka, 51000 Rijeka, Croatia; 2 Faculty of Medicine, University of Rijeka, 51000 Rijeka, Croatia; kristina.skroce@gmail.com; 3 Hospital for Medical Rehabilitation of Heart and Lung Diseases and Rheumatism Thalassotherapia-Opatija, 51410 Opatija, Croatia; 4 Independent Researcher, 11741 Athens, Greece; 5 Department of Social Medicine and Epidemiology, Faculty of Medicine, University of Rijeka, 51000 Rijeka, Croatia; mihaela.marinovic@medri.uniri.hr; 6 School of Nursing and Health Sciences, Hong Kong Metropolitan University, Hong Kong, China; mmkkwok@hkmu.edu.hk

**Keywords:** body composition, exercise therapy, glucagon-like, obesity, sarcopenia

## Abstract

Glucagon-like peptide-1 receptor agonists (GLP-1 RAs) and related incretin-based therapies are highly effective anti-obesity treatments, but weight loss may also include reductions in fat-free mass, raising concern for selected patients at risk of sarcopenia. This narrative review summarizes current evidence and practical, evidence-informed strategies to support muscle health during GLP-1 RA-based anti-obesity therapy. Particular emphasis is placed on identifying higher-risk patients, including older adults, frail individuals, patients with low baseline muscle mass or strength, sarcopenic obesity, chronic kidney disease, low physical activity, or rapid weight loss. Preventive strategies include baseline assessment of body composition and muscle function, individualized resistance and multicomponent exercise, adequate protein intake, attention to nutritional adequacy, and periodic monitoring of body composition, strength, and physical performance. Treatment pace and dose escalation may also be individualized in higher-risk patients when unfavorable changes in fat-free mass or function occur. Current evidence remains limited, and many recommendations are extrapolated from weight-loss, nutrition, exercise, and sarcopenia literature rather than GLP-1 RA-specific trials. Overall, a risk-stratified and multidisciplinary approach may help preserve muscle mass and function while maintaining the cardiometabolic benefits of GLP-1 RA-based therapy.

## 1. Introduction

Glucagon-like peptide-1 receptor agonists (GLP-1 RAs) and related incretin-based therapies, including semaglutide and tirzepatide, have transformed obesity management by producing substantial and sustained weight loss, exceeding that achieved with lifestyle intervention alone. However, as with caloric restriction and bariatric surgery, pharmacologically induced weight loss is not limited to adipose tissue, and a proportion of total weight reduction may derive from fat-free mass (FFM) [1,2,3,4,5,6,7]. This has raised concern that selected vulnerable patients may experience clinically relevant deterioration in skeletal muscle health during treatment [1,4,6,7,8,9,10].

Importantly, loss of FFM is not synonymous with sarcopenia. According to the European Working Group on Sarcopenia in Older People 2 (EWGSOP2), low muscle strength is the primary indicator of probable sarcopenia, while diagnosis is confirmed by reduced muscle quantity or quality; severe sarcopenia is defined by the additional presence of impaired physical performance. Therefore, body composition changes during GLP-1 RA-based therapy should be interpreted together with functional measures such as handgrip strength, chair-rise performance, gait speed, and overall physical performance. The clinical relevance of FFM reduction during GLP-1 RA-associated weight loss remains debated. In many patients, reductions in absolute FFM may partly reflect physiological adaptation to a smaller body size rather than pathological muscle wasting, and improvements in cardiometabolic status, mobility, relative body composition, or muscle quality may occur during weight reduction. Accordingly, the central clinical concern is not FFM reduction per se, but whether changes in body composition are accompanied by reduced strength, impaired physical performance, frailty, or loss of independence, particularly in patients with limited baseline muscle reserve [1,4,5,8,11,12,13].

Risk is not uniform across all patients receiving GLP-1 RA-based therapy. Individuals at greatest risk include older adults, frail patients, those with low baseline muscle mass or strength, sarcopenic obesity, chronic kidney disease (CKD), cancer, chronic inflammatory disease, prolonged immobilization, low habitual physical activity, and those experiencing rapid or marked weight loss [1,5,10,14]. Sarcopenic obesity is particularly relevant because excess adiposity may coexist with impaired muscle strength and function, increasing vulnerability during active weight reduction [1,2,6,8,10,11,12].

Despite the growing body of literature describing changes in body composition during GLP-1 receptor agonist therapy, practical guidance on preventing sarcopenia throughout treatment remains fragmented across obesity, nutrition, exercise, and geriatric medicine. This review addresses that gap by integrating current evidence into a comprehensive clinical framework encompassing risk stratification, baseline assessment, exercise, nutritional interventions, monitoring, and treatment optimization to preserve skeletal muscle during GLP-1–based weight loss. The objective of this review is to provide clinicians with evidence-informed, practical recommendations for minimizing sarcopenia risk while maximizing the metabolic benefits of GLP-1-based anti-obesity therapy.

### 1.1. Methods

This article was designed as a narrative review to integrate current clinical, mechanistic, translational, and guideline-based evidence regarding the prevention of sarcopenia during GLP-1 RA-based anti-obesity therapy. Given the rapidly expanding literature surrounding incretin-based pharmacotherapy and the multidisciplinary nature of muscle preservation during weight loss, encompassing obesity medicine, endocrinology, geriatrics, nutrition, exercise physiology, rehabilitation medicine, body composition analysis, and clinical pharmacology, the primary objective was to synthesize existing evidence into a clinically applicable framework rather than perform quantitative evidence pooling or formal systematic evaluation.

This narrative approach was considered most appropriate because many clinically relevant recommendations—including baseline risk assessment, body composition monitoring, resistance exercise prescription, nutritional interventions, protein supplementation, and individualized treatment optimization—are informed by heterogeneous evidence derived from randomized controlled trials (RCTs), observational studies, mechanistic investigations, expert consensus documents, and international clinical practice guidelines. Accordingly, the review aimed to integrate available evidence into practical recommendations for preserving skeletal muscle mass and function during pharmacologically induced weight loss while identifying current knowledge gaps requiring further investigation.

A targeted literature search was conducted using PubMed and Scopus. To maximize retrieval of relevant publications, manual screening of reference lists from systematic reviews, meta-analyses, clinical practice guidelines, consensus statements, narrative reviews, and original research articles was also performed.

The search included publications from 1 January 2016 through 1 April 2026, corresponding to the period during which major advances occurred in GLP-1 RA pharmacotherapy, obesity management, body composition research, and sarcopenia assessment. Earlier landmark publications published before 2016 were included selectively when considered essential for providing historical context or establishing foundational concepts, including the definitions and diagnostic criteria for sarcopenia, mechanisms of muscle protein metabolism, body composition assessment, resistance exercise physiology, and nutritional strategies relevant to skeletal muscle preservation.

Searches combined Medical Subject Headings, controlled vocabulary, and free-text terms related to obesity pharmacotherapy, GLP-1 receptor agonists, body composition, skeletal muscle, and sarcopenia prevention. Representative search terms included: “GLP-1 receptor agonist”, “GLP-1 RA”, “semaglutide”, “tirzepatide”, “liraglutide”, “obesity”, “anti-obesity pharmacotherapy”, “body composition”, “lean body mass”, “fat-free mass”, “skeletal muscle”, “sarcopenia”, “sarcopenic obesity”, “muscle strength”, “handgrip strength”, “resistance exercise”, “resistance training”, “exercise therapy”, “physical activity”, “protein intake”, “protein supplementation”, “muscle protein synthesis”, “DEXA”, “dual-energy X-ray absorptiometry”, “bioelectrical impedance analysis”, “body composition monitoring”, “nutrition”, “weight loss”, “frailty”, “functional performance”, “clinical guidelines”, and “obesity management”.

Titles and abstracts were initially screened for relevance, followed by full-text evaluation of potentially eligible publications. Articles were considered eligible if they fulfilled one or more of the following criteria:

(i)   evaluated changes in body composition, lean body mass, skeletal muscle mass, or physical function during GLP-1 RA-based anti-obesity therapy;
(ii)  investigated mechanisms contributing to muscle loss during pharmacologically induced weight reduction, including alterations in muscle protein metabolism, anabolic resistance, energy restriction, or body composition;
(iii) evaluated interventions aimed at preserving skeletal muscle during weight loss, including resistance exercise, aerobic or multicomponent exercise, nutritional interventions, protein supplementation, micronutrient optimization, or other supportive strategies;
(iv) examined methods for assessing body composition, muscle strength, physical performance, nutritional status, or sarcopenia risk before or during obesity treatment;
(v)  provided clinical practice recommendations, consensus statements, or guideline-based evidence relevant to obesity management, sarcopenia prevention, exercise prescription, nutrition, or body composition monitoring.

Eligible study designs included RCTs, prospective and retrospective observational studies, cohort studies, case–control studies, cross-sectional investigations, mechanistic and translational studies, systematic reviews, meta-analyses, clinical practice guidelines, consensus statements, and other publications considered relevant to the objectives of this review. Where available, greater emphasis was placed on RCTs, systematic reviews, meta-analyses, and international guideline documents, whereas mechanistic and preclinical investigations were primarily used to support biological plausibility and explain underlying physiological mechanisms. Publications not available in English and studies unrelated to GLP-1-based therapy, obesity, body composition, skeletal muscle, sarcopenia, nutrition, exercise, or muscle preservation were excluded.

The relevance and inclusion of publications were determined through consensus among the authors based on their respective expertise in obesity medicine, clinical pharmacology, nutrition, exercise physiology, rehabilitation, epidemiology, and metabolic health. Discrepancies regarding study inclusion or interpretation were resolved through discussion until consensus was achieved.

Evidence from clinical trials, observational studies, mechanistic investigations, systematic reviews, meta-analyses, and international clinical practice guidelines was synthesized thematically to develop an integrated clinical framework for sarcopenia prevention during GLP-1 RA-based anti-obesity therapy. The narrative synthesis was organized into five interconnected domains: (i) identification of patients at increased risk of sarcopenia and baseline assessment; (ii) lifestyle and exercise interventions for skeletal muscle preservation; (iii) nutritional strategies and monitoring of muscle health during treatment; (iv) body composition and functional monitoring throughout therapy; and (v) individualized pharmacological considerations, including dose titration and optimization of weight-loss pace.

Particular emphasis was placed on integrating evidence from obesity medicine, nutrition, exercise science, geriatrics, and body composition research to formulate practical, evidence-informed recommendations applicable to routine clinical practice. In addition to summarizing current evidence, areas of uncertainty and important knowledge gaps—including limited randomized evidence evaluating combined GLP-1 RA therapy and structured resistance exercise, uncertainty regarding optimal protein intake, absence of validated monitoring intervals, and limited long-term functional outcome data—were identified to provide a balanced interpretation of the available literature.

As a narrative review, this work did not follow the Preferred Reporting Items for Systematic Reviews and Meta-Analyses (PRISMA) reporting framework, and no formal risk-of-bias assessment or quantitative meta-analysis was performed. Although predefined search procedures and eligibility criteria were applied to enhance transparency and reproducibility, substantial heterogeneity in study populations, GLP-1 RA regimens, body composition assessment methods, exercise interventions, nutritional protocols, follow-up duration, and outcome measures precluded formal quantitative synthesis. Furthermore, restriction to English-language publications may have introduced language bias. Nevertheless, a narrative review was considered the most appropriate methodological approach for integrating mechanistic, clinical, and guideline-based evidence into a comprehensive framework for the prevention of sarcopenia during GLP-1-based anti-obesity therapy.

### 1.2. Sarcopenia Risk Identification and Baseline Assessment Prior to GLP-1-Based Therapy

Because GLP-1 RA-based therapies can produce substantial and rapid weight loss, baseline risk stratification may help identify patients most vulnerable to clinically meaningful muscle-related decline. Risk appears highest in individuals with low baseline muscle mass or strength, sarcopenic obesity, frailty, older age, postmenopausal status, type 2 diabetes, CKD, prolonged immobilization, low habitual physical activity, or rapid weight loss. In these patients, reductions in FFM may have greater functional relevance and may warrant closer monitoring, earlier nutritional support, and individualized exercise interventions, particularly resistance training and adequate protein intake [15,16,17,18,19,20,21,22,23,24,25,26,27,28,29,30,31].

In addition to the standard comprehensive evaluation of patients living with obesity—including assessment of comorbidities, detailed laboratory investigations, medication history, lifestyle habits, psychosocial factors, sleep quality, and thorough physical examination—specific baseline assessments targeting body composition, muscle strength, nutrition, and physical activity may be particularly useful prior to initiating GLP-1 RA therapy. Weight reduction achieved with GLP-1-based therapies is not limited to adipose tissue and may be accompanied by clinically relevant losses in FFM, with potential consequences for skeletal muscle strength, physical function, and long-term metabolic health [32,33]. Baseline assessment of body composition is a critical component of GLP-1 therapy initiation, as conventional anthropometric measures such as body weight and body mass index are insufficient to capture qualitative changes in body tissues. DEXA provides a rapid, non-invasive evaluation of fat mass, FFM, and bone mineral density and is widely regarded as the reference method in clinical research. Nonetheless, its limited availability, cost, and requirement for specialized equipment restrict its routine use in clinical practice. In contrast, bioelectrical impedance analysis (BIA) represents a pragmatic alternative, offering a low-cost, non-invasive means of estimating body composition through assessment of total body water derived from tissue electrical resistance. Although the accuracy of BIA may be influenced by assumptions of stable tissue hydration—particularly in individuals with severe obesity or altered fluid distribution—its clinical utility remains high when standardized protocols are applied [34,35,36]. Baseline assessment is useful to identify patients with reduced muscle reserves who may be at heightened risk of disproportionate FFM loss during GLP-1-induced weight reduction. Understanding the technical characteristics, advantages, and limitations of body composition assessment tools is crucial for selecting the appropriate modality. A detailed comparison of DEXA and BIA, including their measurement principles, accuracy, accessibility, and clinical utility, is summarized in Table 1. Consistent longitudinal monitoring using the same assessment modality enables early detection of unfavorable changes in FFM and facilitates timely, targeted interventions to preserve skeletal muscle function and maximize the metabolic benefits of GLP-1 therapy [37]. Evaluation of muscle strength and function is an important complement to body composition assessment when initiating and monitoring GLP-1-based therapy. While DEXA and BIA provide structural insight into muscle mass, functional measures capture clinically relevant physical capacity that directly impacts patient outcomes. Isokinetic testing is considered the gold standard for muscle strength assessment but is often impractical in routine clinical settings. Handgrip strength, assessed with a dynamometer, offers a simple, validated alternative strongly correlated with total body muscle mass and overall strength. Additionally, the chair stand test evaluates lower extremity strength, balance, and neuromuscular coordination, essential for daily mobility tasks. Together, these functional assessments enable early identification of patients at risk of functional decline and support targeted muscle-preserving interventions throughout GLP-1 therapy [38]. Comprehensive baseline and follow-up assessments of nutrition and physical activity are essential to contextualize changes in muscle mass and function during GLP-1 therapy. Nutritional assessment involves quantifying total energy intake and macronutrient composition, with particular focus on protein consumption measured in grams per kilogram of body weight. Evaluating micronutrient status—especially vitamin D, calcium, and omega-3 fatty acids—is integral due to their roles in muscle metabolism. Dietary intake is typically assessed via 24 h recalls, food frequency questionnaires, or detailed dietary records, collected at baseline and monitored periodically to detect changes induced by appetite suppression or dietary modification [38,39]. Physical activity evaluation encompasses detailed documentation of exercise type (resistance, aerobic, balance), frequency, intensity, and duration. Validated instruments such as the International Physical Activity Questionnaire or wearable activity trackers provide objective measures of habitual physical activity patterns. Initial assessment establishes baseline activity levels, while follow-up evaluations at 3-month intervals identify behavioral changes affecting muscle health. Together, these assessments provide critical data to interpret body composition and functional outcomes throughout treatment [38,39,40].

### 1.3. Lifestyle and Exercise Interventions for Sarcopenia Prevention

#### 1.3.1. Lifestyle Counseling as a Foundational Preventive Strategy

Lifestyle counseling can support adoption of healthier dietary and physical activity behaviors [41]. For patients undergoing GLP-1-based anti-obesity therapy, lifestyle counseling represents a foundational preventive strategy, aiming not only to achieve weight reduction but also to preserve skeletal muscle mass and prevent sarcopenia. Evidence suggests that intentional incorporation of resistance and strength training exercises, performed at least two to three times per week and targeting major muscle groups, can attenuate FFM loss and maintain functional capacity during pharmacologically induced weight loss [42]. In addition to structured exercise, patients should be encouraged to increase overall physical activity, such as daily walking, cycling, or stair climbing, to enhance energy expenditure and support cardiovascular and musculoskeletal health. Nutritional interventions are equally critical; dietary counseling should emphasize sufficient protein intake—ideally 1.2–1.6 g/kg body weight per day—distributed evenly across meals, while ensuring total caloric intake is adequate to prevent excessive catabolism of muscle tissue [43]. Micronutrient sufficiency, particularly vitamin D and calcium, may further support muscle and bone health, especially in older adults [44]. Clinicians should also provide education on realistic expectations for body composition changes, highlighting that reductions in fat mass are prioritized while preservation of FFM is important for maintaining strength, metabolic health, and long-term functional independence [45]. Social support may improve adherence to lifestyle interventions and help sustain behavioral change [46]. Consistent with the evidence, a multidisciplinary strategy combining pharmacological treatment, lifestyle changes, and social support may help maintain FFM, support functional independence, and promote favorable long-term outcomes for patients receiving GLP-1-based anti-obesity therapy.

#### 1.3.2. Resistance Exercise as the Cornerstone Intervention

Substantial weight reduction achieved with GLP-1 RAs may include reductions in FFM, particularly among patients with low baseline muscle reserve, older age, or limited habitual physical activity. Resistance training (RT) provides an anabolic stimulus and is supported by evidence from caloric restriction, obesity, aging, and sarcopenia studies as a strategy to attenuate FFM loss and improve strength. However, direct interventional evidence in GLP-1 RA-treated populations remains limited; therefore, RT should be framed as an evidence-informed, risk-stratified strategy rather than an established GLP-1 RA-specific standard. In practice, progressive RT may be considered 2–3 times weekly, targeting major upper- and lower-body and trunk muscle groups with progressive overload. Training may use free weights, machines, resistance bands, or bodyweight exercises, with lower starting intensity and slower progression for frail or deconditioned patients. Aerobic activity may be added for cardiometabolic health, while RT remains the principal exercise modality for muscle preservation. Supervised or professionally guided programs may be useful for patients with frailty, comorbidities, low baseline strength, or poor exercise confidence [26,42,47,48,49,50,51,52,53,54,55,56,57,58,59].

#### 1.3.3. Role of Aerobic and Multicomponent Physical Activity

**Figure 1:**
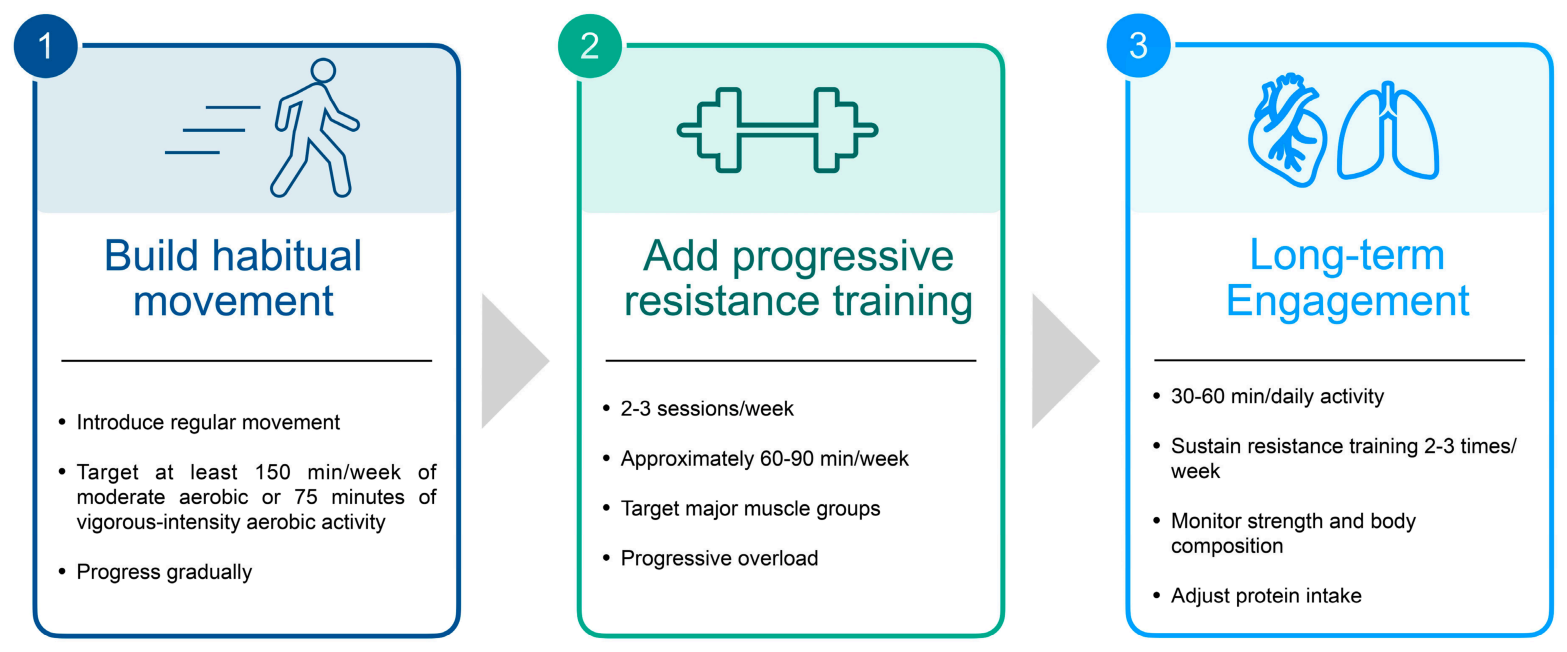
Stepwise exercise framework for patients receiving GLP-1-based anti-obesity therapy. Note: A practical three-step exercise framework for supporting muscle preservation during pharmacologically induced weight loss: (**1**) building habitual movement through gradual increases in regular activity and progression toward recommended aerobic activity targets; (**2**) adding progressive resistance training 2–3 times per week, targeting major muscle groups with progressive overload; and (**3**) maintaining long-term engagement through sustained aerobic activity and resistance training, with ongoing monitoring of strength, FFM, and nutritional adequacy.

While RT represents the primary strategy to preserve skeletal muscle mass during weight loss, aerobic and multicomponent physical activity play a complementary role in optimizing cardiometabolic health, physical function, and long-term weight maintenance [60,61]. Direct comparisons reveal that GLP-1 RAs generally produce greater short-term weight loss than exercise alone, but exercise may be more effective for preserving FFM and cardiorespiratory fitness [62,63,64]. Randomized and real-world evidence suggests that combining GLP-1 RA therapy with aerobic exercise (with or without combined resistance exercise) yields additive benefits, including greater reductions in metabolic syndrome severity, abdominal obesity, oxidative stress and inflammation, and improved weight loss maintenance after cessation of pharmacotherapy [63,65]. Moreover, in contrast to pharmacotherapy, exercise is a low-cost intervention and represents a behavioral change that, in principle, can be continued in a real-world setting after termination of the supervised treatment [64]. In patients receiving GLP-1-based therapy, moderate aerobic exercise can enhance cardiometabolic improvements achieved through pharmacological weight loss without substantially increasing the risk of unfavorable FFM loss, particularly when combined with RT and adequate protein intake. This conclusion is supported by emerging clinical evidence that combining physical activity with pharmacological weight-loss therapy produces additive improvements in body weight, glycemic measures, and other cardiometabolic markers compared with pharmacotherapy or exercise alone [66]. In order to optimize the outcomes of both aerobic and resistance exercise while enhancing the positive effects of GLP-1 RA therapy, a practical three-step approach proposed by the World Health Organization (WHO) [61], American College of Sports Medicine [67], American Diabetes Association [68], and European Association for the Study of Obesity [69] guidelines may be used to inform a practical approach. It recommends (1) introducing regular movement gradually, targeting 150 min of moderate-intensity or 75 min of vigorous aerobic activity per week [70]; (2) incorporating RT for 60–90 min weekly, using accessible methods such as resistance bands, weights, or bodyweight exercises; and (3) sustain long-term engagement with 30–60 min of daily aerobic activity alongside RT 2–3 times weekly (see Figure 1). Beyond aerobic conditioning, multicomponent exercise programs [54] incorporating balance and flexibility, and functional training are particularly relevant for individuals with obesity receiving GLP-1-based therapy who are at risk of sarcopenia, especially older adults and those with reduced baseline physical capacity. In this population, preservation of muscle function is as important as preservation of muscle mass.

Balance training involves exercises designed to improve stability and coordination, yielding significant biological effects across various body systems. This training enhances neuromuscular control by improving communication between the nervous system and muscles, which boosts proprioception, coordination, and reaction times, thereby reducing fall risk. Moreover, balance training engages multiple muscle groups, strengthening stabilizer muscles and promoting overall muscular development and endurance [71,72]. On the other hand, flexibility and mobility exercises support joint range of motion, movement efficiency and contribute to the maintenance of functional independence in older adults [73]. It is important to underline that exercise interventions in patients receiving GLP-1 therapy should not follow a uniform template but should be individualized. Training variables, including modality, intensity, volume, frequency, progression, and recovery, should be tailored to the patient’s age, comorbidities, baseline muscle mass, functional capacity, and rate of weight loss. A structured, personalized approach may help optimize cardiometabolic benefits while minimizing FFM loss, injury risk, and treatment-associated fatigue. Exercise prescription during GLP-1-based therapy should also account for patient differentiation. For example, older adults, frail individuals, and patients with low baseline muscle strength may require lower initial training loads, closer supervision, and slower progression to reduce injury risk while still providing sufficient anabolic stimulus. Patients with low habitual physical activity may benefit from a gradual transition from basic movement goals toward structured resistance training, whereas individuals with higher baseline fitness may tolerate earlier progression in training volume and intensity. Therefore, resistance and multicomponent exercise interventions should be individualized accordingly.

In order to optimize the outcomes of both aerobic and resistance exercise while enhancing the positive effects of GLP-1s, a practical three-step approach may be followed: (1) building habitual movement and progressing toward recommended aerobic activity targets; (2) incorporating progressive resistance training; and (3) maintaining long-term engagement with combined aerobic and resistance exercise, while monitoring strength, FFM, and nutritional adequacy (see Figure 1).

### 1.4. Nutritional Strategies and Monitoring of Muscle Health During GLP-1-Based Therapy

#### 1.4.1. Protein Intake Recommendations During GLP-1-Based Therapy

GLP-1 RAs promote marked weight loss but may reduce FFM, supporting consideration of targeted protein intake to preserve skeletal muscle and mitigate sarcopenia. Several guidelines and expert statements propose 1.2–1.6 g/kg/day (up to 2.0 g/kg in high-risk cases), calculated on ideal or adjusted body weight, to support muscle protein synthesis (MPS) during caloric deficits. Even distribution across meals optimizes anabolic responses, while monitoring may help identify appetite-suppression-induced shortfalls [74,75,76]. Elevated protein needs during GLP-1 RA therapy exceed the recommended daily allowance (RDA; 0.8 g/kg/day) due to heightened catabolism and anabolic resistance; 1.2–1.6 g/kg/day preserves FFM, with studies showing ~40–60% reduction in FFM loss versus lower intakes. For a 90 kg patient targeting 70 kg ideal weight, this equates to 84–112 g/day, prioritizing high-quality sources (leucine > 2.5 g/meal) like whey (bioavailability score 1.0), eggs, fish, and soy to maximize MPS stimulation. Adjustments for age (>65 years: 1.6–2.0 g/kg), comorbidities (CKD: plant-based to limit phosphorus), and baseline sarcopenia risk ensure personalization, as FFM-referenced dosing (1.6–2.3 g/kgFFM) better predicts preservation in obesity [75,76,77,78,79].

Protein and nutritional strategies should be individualized rather than applied uniformly. Older adults, frail patients, postmenopausal women, and individuals with low baseline muscle reserve may require closer attention to protein adequacy during GLP-1-associated appetite suppression. In patients with type 2 diabetes, preserving muscle quality is relevant for metabolic health, whereas in CKD, protein targets should be adapted to renal function and clinical status. Thus, nutritional monitoring should consider not only protein quantity, but also safety, tolerability, comorbidities, and feasibility for the individual patient.

Skewed intake (e.g., >50% at dinner) yields suboptimal MPS; evenly distributing 25–40 g/meal across 3–4 occasions boosts 24 h MPS by 25%, with refractory period minimization via repeated leucine pulses. Crossover trials confirm 30 g/meal (breakfast/lunch/dinner) outperforms pulsed patterns, enhancing net protein balance during energy restriction akin to GLP-1 RA effects. Snacks (10–15 g, e.g., Greek yogurt) bridge gaps, sustaining elevated plasma essential amino acids (EAAs) for satellite cell activation and hypertrophy signaling [75]. GLP-1 RAs reduce hunger/satiety signals, slashing total intake by 20–30%, with protein often <1.0 g/kg despite intentions (only 43% achieve 1.2 g/kg). Micronutrient gaps compound risks, underscoring shakes/supplements (20–30 g/serving) when solid food tolerance wanes. Weekly tracking via apps (e.g., MyFitnessPal), dietitian consults, and biomarkers (e.g., 3-methylhistidine for catabolism) enable adherence, with >75% self-reporting increased protein post-initiation, but objective shortfalls are prevalent [74,77]. Data affirm that multimodal nutrition preserves function [80]. Practical implementation should focus on individualized protein targets, meal distribution, tolerability, renal function, dietary preferences, and reassessment when appetite suppression limits adequate intake [74,75,76,77,78,80,81]. Combining protein intake recommendations with RT, performed 2–3 times per week, yields additive effects on muscle preservation during GLP-1 RA therapy by enhancing MPS and counteracting anabolic resistance. Adjunctive nutritional strategies, including β-hydroxy-β-methylbutyrate (HMB) and omega-3 fatty acids, remain under investigation, and their role in preventing sarcopenia during GLP-1 RA-based therapy has not yet been established. Similarly, precision nutrition approaches based on biomarkers or metabolomic profiling remain investigational and should be considered future research directions rather than established clinical strategies [78,80,81].

#### 1.4.2. Protein Quality, Timing, and Supplementation

GLP-1 therapies are expected to be increasingly used for the long-term treatment of obesity, with the WHO adopting its first guideline in December 2025 [82]. Protein intake should be tailored to adult patients’ nutritional needs in light of suppressed appetite and muscle loss induced by GLP-1 therapies for sarcopenia prevention [78]. Adequate high-quality protein (including leucine) is important to preserve muscle during rapid weight reduction when using obesity medications like GLP-1 RAs. According to the joint Advisory (August 2025) from four American organizations in lifestyle medicine, nutrition, and obesity, consuming 1.2–1.6 g of protein per kilogram of body weight per day (g/kg/day) is proposed during active weight loss, considering clinical trials on GLP-1 RAs showed that FFM or lean soft tissue has accounted for ~10–25% of total weight reduction (possibly greater in men than women) [83]. The higher protein intake is aligned with the Dietary Guidelines for Americans 2025–2030 released in January 2026, which has shifted from the previous RDA of 0.8 g/kg/day [84]. Nevertheless, GLP-1 RAs generally reduced macronutrient (including protein) intake due to appetite suppression, and patients receiving GLP-1 therapies may not reach the proposed protein target as reported in a small cross-sectional study [77]. Prioritizing protein quality becomes equally important as protein quantity. High-quality proteins rich in all nine EAAs are crucial for preserving muscle mass by balancing the cycle of MPS and muscle protein breakdown (MPB). Leucine, one of the three branched-chain amino acids (BCAAs) as part of all nine EAAs, is hypothesized to act as the trigger for MPS by directly activating the mechanistic target of rapamycin complex 1 (mTORC1) signaling pathway [85]. Together with all other eight EAAs, they serve as the building blocks for maintaining muscle mass when synthesis balances with breakdown, rather than BCAAs or leucine alone [86]. Other macronutrients, vitamins, and minerals also play a role in MPS [87,88]. Whole foods (high in EAAs bioavailability and low in saturated fat) from animal sources (e.g., fish, seafood, poultry, lean meat, eggs, and dairy) and plant sources (beans, peas, lentils, nuts, seeds, and soy products) are generally recommended over isolated protein sources (whey, casein, soy protein) for comprehensive nutrition in otherwise healthy adults without renal impairment [89]. Timing of protein intake to maximize muscle growth has been investigated, giving rise to the “anabolic window” concept emphasizing protein and carbohydrate intake within a narrow 30 min–2 h post-exercise period [90]. However, studies on RT found similar muscular response between pre- and post-exercise protein intake [91]. MPS has also been demonstrated to remain sensitive to protein intake for at least 24 h after RT [92]. As such, consuming protein both pre- and post-exercise within a 4–6 h period has been suggested to leverage synergism between exercise and amino acid availability to optimize muscle hypertrophy [93]. Further, total protein intake, spread evenly across daily meals, may be more effective for muscle anabolism than skewed intake [94]. To meet the protein target of e.g., 1.6 g/kg/day, ~25–40 g of proteins per meal across a minimum of four meals (~3–4 h apart) has been recommended [95,96]; however, it remains unclear whether protein targets for patients with obesity should be based on actual, ideal, or adjusted body weight to avoid overestimating needs [83]. Given the appetite-suppressant effects of GLP-1 therapies, meeting the protein target can be challenging through whole foods alone. Protein supplements (e.g., protein powders/drinks including whey, casein, and plant-based) may be considered when dietary intake is inadequate [79]. Future long-term RCTs are warranted to assess the effects of dietary supplements combined with GLP-1 therapies on nutritional status, muscle mass and strength, and metabolic health.

#### 1.4.3. Micronutrients and Adjunct Nutritional Strategies

Multivitamin and mineral supplements may be proactively considered to address potential nutritional deficiency in key nutrients (such as vitamin D, calcium, vitamin B12) among patients receiving GLP-1 therapies for obesity, with types and doses tailored to individual nutritional needs [83]. Nutritional deficiencies are not uncommon among patients on GLP-1 therapies. Two-thirds of GLP-1 users were reported to have inadequate calcium, iron, and vitamin D intake [97]. Particularly, vitamin D insufficiency was observed in ~12% and 22% of GLP-1 users at 6 and 12 months respectively based on a retrospective healthcare claims study [98]. Adequate vitamin D is important for muscle function [99,100]. Vitamin D deficiency is a recognized risk factor for sarcopenia, and supplementation is recommended when baseline levels are low to support strength and reduce fall risk [101,102].

Further, omega-3 polyunsaturated fatty acids, especially eicosapentaenoic acid and docosahexaenoic acid, have been suggested to support muscle function, primarily by promoting MPS and reducing MPB via mechanistic target of rapamycin-related and nuclear factor kappa B signaling pathways respectively [103]. Evidence from meta-analyses of RCTs is currently mixed. One study found omega-3 fatty acids supplementation may result in very small increases in muscle strength but no change in muscle mass and function in healthy young and older adults [104]. Another study found omega-3 fatty acids supplementation together with RT improved muscle strength, albeit not muscle mass [105]. A study also found that omega-3 fatty acids supplementation increased whole-body protein synthesis rate, albeit not muscle-specific ones [106]. To what extent omega-3 fatty acids enhance muscle anabolic sensitivity to amino acids requires future investigation. Creatine is naturally found in myocytes for energy metabolism. Creatine supplementation has been used to promote muscle strength and enhance exercise performance by facilitating rapid regeneration of adenosine triphosphate (ATP) during exercise [107]. A meta-analysis of RCTs demonstrated creatine supplementation increased FFM for all age groups, particularly with RT [108]. Creatine supplementation is generally safe to consume, as indicated by a meta-analysis of observational and experimental designs showing a modest, transient increase in serum creatinine, which was likely due to metabolic turnover rather than renal impairment [109]. Taken together, the effects of creatine, omega-3, and vitamin D supplementation in preventing sarcopenia remain unstudied in patients receiving GLP-1 therapies for obesity [79].

### 1.5. Body Composition and Functional Monitoring During Treatment

Because some weight loss during GLP-1 RA therapy may include FFM, periodic assessment of body composition and physical function may be useful, particularly in patients with frailty, low baseline muscle reserve, CKD, type 2 diabetes, low physical activity, or rapid weight loss. Dual-energy X-ray absorptiometry (DEXA) and magnetic resonance imaging provide accurate body composition assessment, but cost and availability may limit routine use. Bioelectrical impedance analysis (BIA) can be used pragmatically for serial outpatient monitoring if the same device and protocol are used and results are interpreted in light of hydration status and population-specific prediction equations [26,50,110,111,112].

Because muscle mass alone does not define sarcopenia, monitoring should include feasible functional measures. According to EWGSOP2, low muscle strength is the primary indicator of probable sarcopenia, with diagnosis confirmed by low muscle quantity or quality and severity defined by impaired physical performance. Practical measures may include handgrip strength, gait speed, chair-rise testing, and patient-reported mobility. Reassessment every 3 months or so may be reasonable in higher-risk patients; however, optimal intervals are not established. Disproportionate declines in FFM, strength, or physical performance may prompt review of RT participation, aerobic exercise volume, protein intake, total energy intake, and treatment pace [26,57,80,113,114,115].

### 1.6. GLP-1 Dose Titration and Weight-Loss Pace Optimization

**Figure 2:**
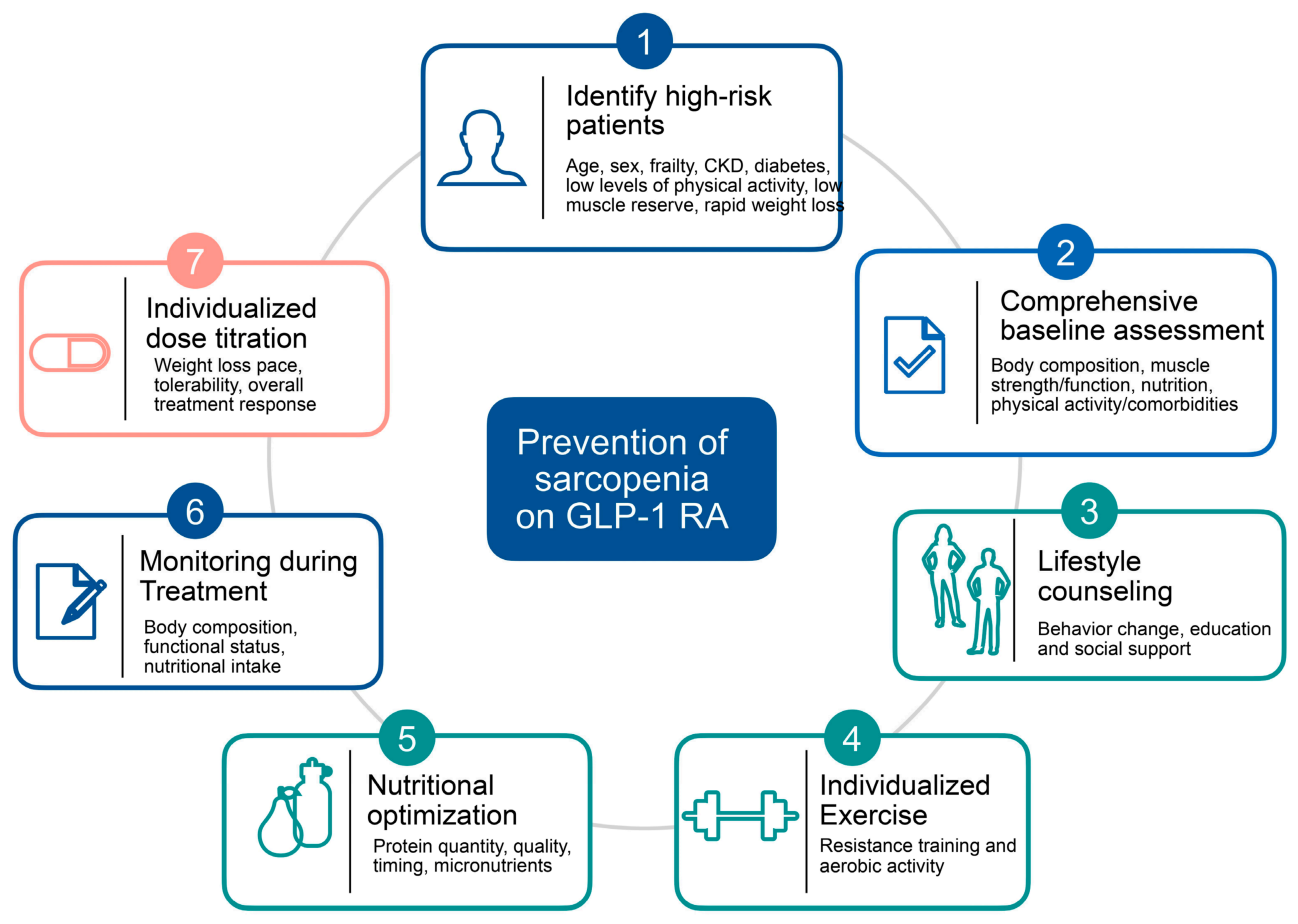
Multicomponent clinical framework for the prevention of sarcopenia in patients receiving GLP-1 RA therapy. Note: A stepwise, risk-stratified approach to minimize clinically relevant FFM loss and functional decline during GLP-1 therapy, including: (**1**) identification of high-risk patients according to age, sex, baseline muscle mass and strength, frailty, CKD, diabetes, physical activity level, and weight-loss pace; (**2**) comprehensive baseline assessment; (**3**) lifestyle counseling; (**4**) individualized resistance and multicomponent exercise; (**5**) nutritional optimization according to risk and comorbidities; (**6**) monitoring of body composition, muscle strength, physical performance, and nutritional status; and (**7**) individualized dose titration based on patient response.

Standard GLP-1 RA titration schedules in product labeling and clinical guidelines remain the primary framework for dose escalation. In higher-risk patients, clinicians may also consider weight-loss pace, dietary intake, physical activity, functional status, and body composition trends when deciding whether to escalate therapy. If rapid weight loss or disproportionate decline in FFM, strength, or physical performance is observed, maintaining the current dose temporarily while optimizing nutrition, RT, and multidisciplinary support may be reasonable. This approach is based mainly on clinical judgment and indirect evidence rather than GLP-1 RA-specific trials, and should therefore be interpreted as an individualized clinical consideration rather than an established standard. A comprehensive, multicomponent clinical framework is summarized in Figure 2.

## 2. Discussion

**Figure 3:**
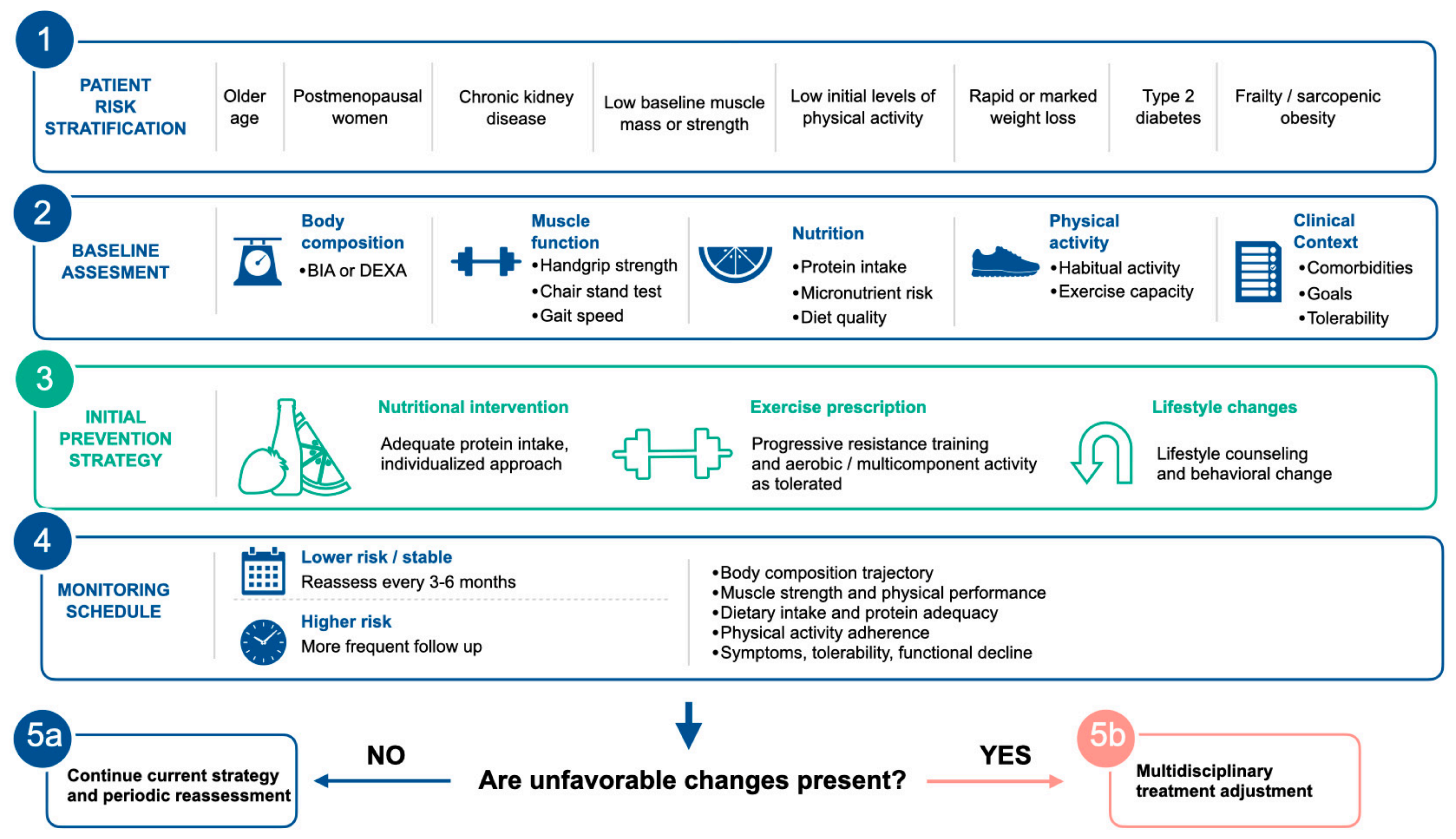
Practical clinical algorithm for prevention of sarcopenia during GLP-1-based anti-obesity therapy. Note: The algorithm summarizes a practical clinical pathway for minimizing clinically relevant loss of skeletal muscle mass and function during GLP-1-based anti-obesity therapy. It integrates patient risk stratification, baseline assessment, nutritional intervention, exercise prescription, lifestyle modification, monitoring schedule, and treatment adjustment. Monitoring may include body composition trajectory, muscle strength and physical performance, dietary intake and protein adequacy, physical activity adherence, treatment tolerability, and signs of functional decline. If unfavorable changes occur, nutritional support, exercise prescription, weight-loss pace, dose escalation, and multidisciplinary management may be reassessed.

A multidisciplinary care approach is important for preventing sarcopenia in patients undergoing GLP-1 RA therapy for obesity, as it integrates expertise from endocrinologists, registered dietitians, physical therapists, and geriatric specialists to address the complex interplay of pharmacologic weight loss and preservation of skeletal muscle mass and function. Tailoring preventive strategies according to individual risk profiles, treatment responses, and patient preferences may enhance both efficacy and long-term adherence, while systematically incorporating sarcopenia prevention measures may improve the safety profile and sustainability of GLP-1 RA therapy. Such collaborative frameworks mitigate the multifaceted risks associated with disproportionate FFM loss, ultimately fostering comprehensive cardiometabolic and functional health improvements (Figure 3).

However, the available evidence should be interpreted with caution. Although reductions in fat-free mass have been reported during GLP-1 RA-associated weight loss, this does not directly imply that all patients develop clinically meaningful sarcopenia or functional decline. The main concern is therefore not FFM loss per se, but whether this loss translates into impaired muscle strength, reduced physical performance, frailty, or poorer long-term outcomes, particularly in individuals with low baseline muscle reserve or increased vulnerability to functional decline. In patients at increased risk of low muscle reserve or functional decline, involvement of clinicians, dietitians, and exercise professionals may help contextualize changes in nutritional intake, physical activity, muscle strength, and physical performance during treatment [5,116,117].

An important controversy is how FFM reductions during GLP-1 RA-induced weight loss should be interpreted. Loss of absolute FFM may occur alongside substantial fat mass reduction, improved cardiometabolic status, reduced mechanical loading, and improved mobility. In this context, reductions in FFM may partly reflect physiological adaptation to a smaller body size rather than pathological muscle wasting. Moreover, fat-free mass is not identical to skeletal muscle. This balanced interpretation avoids overstating the risk of sarcopenia while still recognizing that selected high-risk patients may require closer attention during pharmacological weight loss.

Patient preferences significantly influence strategy selection and adherence; therefore, supportive strategies should remain feasible, acceptable, and proportional to individual risk rather than uniformly prescribed. Adherence may improve when interventions align with patient preferences, lifestyle, and cultural dietary patterns [5,8].

Although preservation of muscle function may plausibly support long-term mobility, quality of life, metabolic health, and weight-loss maintenance, the magnitude of these benefits in GLP-1 RA-treated populations remains insufficiently established. Claims regarding specific reductions in hospitalization, healthcare expenditure, or functional decline require GLP-1-specific prospective evidence before they can be considered established [116,117,118].

Digital tools may support monitoring and adherence in the future, but their role in preventing sarcopenia during GLP-1 RA therapy remains unproven [116,119].

Similarly, reimbursement models, electronic medical record alerts, and implementation-science frameworks are important health-system considerations, but they extend beyond the current evidence base on GLP-1 RA-associated body composition changes. These topics are therefore more appropriately addressed as future implementation challenges rather than established clinical recommendations [5,116].

Patient education remains relevant, but the message should be balanced: GLP-1 RA therapy can produce substantial cardiometabolic benefits, reductions in FFM are not automatically equivalent to clinically meaningful sarcopenia, and preservation of strength and physical function should be considered alongside weight reduction [116,119].

Several important limitations of the current evidence should be acknowledged. First, despite the biological rationale supporting resistance exercise during GLP-1-based weight loss, few RCTs have specifically evaluated the effects of combining GLP-1 receptor agonists with structured resistance exercise on the preservation of skeletal muscle mass, strength, and physical function, and even fewer have assessed these outcomes as predefined primary endpoints. Second, although higher protein intake is commonly recommended during weight loss to support muscle preservation, the optimal amount, distribution, and protein source for individuals receiving GLP-1-based therapy have yet to be established. Third, no evidence-based consensus exists regarding the optimal frequency or modality for monitoring body composition and muscle function during treatment. Finally, although GLP-1 receptor agonists have demonstrated substantial benefits for weight reduction and cardiometabolic health, data on long-term functional outcomes—including muscle strength, physical performance, frailty, falls, and disability—remain limited. These evidence gaps highlight the need for adequately powered prospective RCTs to define evidence-based strategies for preserving skeletal muscle while maintaining the metabolic benefits of GLP-1-based anti-obesity therapy.

Future prospective studies should clarify which patients are most vulnerable to clinically meaningful functional decline and whether multimodal interventions combining resistance training, nutritional support, and structured monitoring can prevent adverse muscle-related outcomes during GLP-1 RA therapy.

The existing evidence is characterized by substantial heterogeneity in study designs, patient populations, and outcome measures. While this narrative review aimed to timely provide a comprehensive qualitative synthesis, a formal grading approach was not performed. A systematic review incorporating evidence grading should be conducted in the future, when more human evidence from rigorous observational cohort studies and RCTs becomes available.

## 3. Conclusions

GLP-1 RA-based therapies provide substantial benefits for obesity management, but selected high-risk patients may require attention to muscle mass, strength, and physical performance during treatment. This review summarizes evidence-informed, risk-stratified strategies, including baseline assessment, resistance exercise, adequate protein intake, nutritional monitoring, functional assessment, and individualized treatment adjustment. Future GLP-1 RA-specific studies are needed to define the most effective approaches for preserving muscle-related outcomes.

## Figures and Tables

**Table 1:** Comparison of DEXA and BIA for body composition assessment.

Characteristic	Dual-Energy X-Ray Absorptiometry (DEXA)	Bioelectrical Impedance Analysis (BIA)
**Measurement principle**	X-ray attenuation differentiates bone, fat, and lean tissue	Electrical impedance measures total body water to estimate lean and fat mass
**Parameters assessed**	Total and regional fat mass, fat-free mass, bone mineral density	Total body water, fat mass, fat-free mass (via predictive equations)
**Accuracy**	High; considered gold standard in clinical research	Moderate; accuracy affected by hydration status and population-specific equations
**Equipment requirements**	Specialized, costly radiology equipment	Portable, low-cost devices
**Accessibility**	Limited to specialized centers	Widely available, suitable for routine clinical use
**Duration and convenience**	10–20 min; requires trained personnel	<5 min; easy to operate
**Limitations**	Exposure to low-dose radiation; high cost; limited availability	Sensitive to hydration changes, recent exercise, food intake; less accurate in severe obesity
**Use in Obesity**	Accurate	Reduced due to altered hydration and tissue distribution
**Clinical Utility**	Ideal for precise assessment and research	Practical for screening and longitudinal monitoring

## Data Availability

No new data were created or analyzed in this study. Data sharing is not applicable to this article.

## Abbreviations

The following abbreviations are used in this manuscript:

ATP	adenosine triphosphate
BCAAs	branched-chain amino acids
BIA	bioelectrical impedance analysis
CKD	chronic kidney disease
DEXA	dual-energy X-ray absorptiometry
EAAs	essential amino acids
EWGSOP2	European Working Group on Sarcopenia in Older People 2
FFM	fat-free mass
GLP-1 RA	glucagon-like peptide-1 receptor agonist
GLP-1 RAs	glucagon-like peptide-1 receptor agonists
HMB	β-hydroxy-β-methylbutyrate
MPS	muscle protein synthesis
MPB	muscle protein breakdown
mTORC1	mechanistic target of rapamycin complex 1
PRISMA	Preferred Reporting Items for Systematic Reviews and Meta-Analyses
RCT	randomized controlled trial
RCTs	randomized controlled trials
RDA	recommended daily allowance
RT	resistance training
WHO	World Health Organization
